# The American Dental Association

**Published:** 1891-07

**Authors:** 


### EDITORIAL.

### The American Dental Association.

The annual meeting of this body takes place in Saratoga, N. Y., on the first Tuesday of August, continuing four days. This society was organized over thirty years ago. It was organized by, and consisted of delegates from the various States. and local societies of the United States. It is regarded as the representative body of the profession of this country. It has accomplished great good for the profession in many ways, but especially by its efforts more pronouncedly put forth in the early years of its existence for the organization and maintenance of State and local societies. It was instrumental, in the early years of its existence, in securing the organization of a large number of dental societies, nearly all of which remain to the present time and have done good work for the profession. Within the last ten or twelve years, however, this central organization has relaxed its effort in this direction and less has been accomplished relatively during this time than before. There is, however, apparent a revival in this respect; there is in the minds of many the idea that the association should take hold of this work with increased energy. There is much that could be done by properly directed effort. There are many parts of the country where new societies should be organized and doubtless much could be done, at least suggestively, by this body in the way of improving and expanding dental society work. So much has uniformity and routine methods of procedure been employed in dental society work that it has often become wearisome.

Would it not be well to have a committee of a proper number, and of suitable persons, whose duty it should be to take this subject into consideration with power to suggest and co-operate with local societies with a view of increasing interest, stimulating enthusiasm, devising new methods of work, and increasing the efficiency of the old.

Dental societies are the practitioners' colleges, and it is as important that they should be maintained and made most efficient as for any other arm of the professional service.

Another direction in which this body has been of great service is that of the dental education. It has had, from the beginning, its annual report upon this subject. These have usually been valuable productions giving important suggestions, and doubtless influencing the profession, and especially those engaged in educational matters in a very marked degree.

The declarations that have been made in this body in the way of papers, discussions and resolutions have always been in the direction of progress, and have in many instances exercised a decided influence for good.

The professional ethics have not been overlooked. A code of ethics of a very high order was devised and adopted by this body in its early history, and this was to be, and has been, not only for the regulation of the professional conduct of the members of this body, but for those of all societies who have membership in this organization. The influence of this code in raising and stimulating true professional character and dignity can not be estimated.

Dental legislation has also received the sanction of this body, and while perhaps it might have done more in this direction than it has, yet whenever the occasion has occurred to make a declaration, it was for the enactment and maintenance of good wholesome regulating legislation. While the things here mentioned have received attention the art and science of the profession have both had a large share of attention and consideration, and perhaps occasionally more relatively, than they should have received. Now should not every true professional dentist, every well wisher of his chosen calling support and co-operate with an organization seeking to accomplish the work here outlined?

Every society should have its representatives in this body and have a part in the great work it is seeking to accomplish. This would be a stimulus to all societies to seek the elevation of the profession within the sphere of their influence.

